# Personalized supervised and unsupervised intracranial sleep decoding during deep brain stimulation

**DOI:** 10.1038/s41746-026-02368-0

**Published:** 2026-01-22

**Authors:** Clay Smyth, Md Fahim Anjum, Jin-Xiao Zhang, Jiaang Yao, Reza Abbasi-Asl, Philip Starr, Simon Little

**Affiliations:** 1 Department of Bioengineering, University of California, San Francisco, San Francisco, CA USA; 2 Department of Neurology, University of California, San Francisco, San Francisco, CA USA; 3 Department of Neurosurgery, University of California, San Francisco, San Francisco, CA USA

**Keywords:** Biomedical engineering, Neurological disorders, Computational neuroscience, Data processing, Machine learning

## Abstract

Impaired sleep in Parkinson’s Disease (PD) is a significant unmet need. Targeting sleep stage-specific neurophysiologies with adaptive Deep Brain Stimulation (aDBS) may ameliorate sleep disruption. This study analyzes the efficacy of personalized machine learning approaches on classifying sleep stages from participants receiving deep brain stimulation. We acquired 283 hours of multi-night intracranial cortico-basal recordings with synchronized sleep stage labels derived from scalp EEG across 5 participants during chronic stimulation. Five-stage classification accuracy across PD subjects averaged 80.2% (±0.9% SEM). When constraining sleep classification to algorithms implementable in currently available DBS devices, e.g., binary NREM classification using linear models, an average accuracy of 85.9% (±0.4% SEM) was achieved for PD subjects. Additionally, linear models trained on unsupervised cluster labels achieved an average accuracy of 83.5% (±5.6% SEM) when discriminating NREM sleep. Overall, this demonstrates the feasibility of personalized supervised and unsupervised ML models for sleep classification using intracranial data during stimulation. The Institutional Review Board approved the parent study protocol, and the study was registered on clinicaltrials.gov (NCT0358289; IDE G180097).

## Introduction

Parkinson’s disease (PD) was initially formulated as a disorder of movement. However, more recently, PD has been understood as a broader neuropsychiatric disorder, with greater emphasis placed on non-motor symptoms^1^. Sleep dysfunction, and its consequences, are of particular importance because they are common, disabling, and difficult to treat in PD^2–4^.

Sleep is commonly divided into four stages: rapid eye movement (REM), which physiologically resembles wakefulness; and non-rapid eye movement sleep (NREM), divided into three stages of increasing depth and delta power (N1-3)^5^. Sleep disruption in people with Parkinson’s disease (PwP) usually involves reduced time in, and increasingly fragmented, NREM (particularly N2/N3) sleep^6^. REM sleep behavior disorder is also prevalent, which includes loss of normal atonia during REM and resultant acting out of dreams^6^. Chronic sleep disruption in PwP reduces quality of life and increases caregiver burden^7,8^. Reduced N3 sleep (or slow wave sleep; SWS) also correlates with the progression of motor symptom severity^9,10^. Ameliorating the consequences of sleep dysfunction in PD is a high priority because of the role of sleep for glymphatic clearance of protein aggregates and maintaining cognitive capacities^11–15^. Therefore, addressing sleep dysfunction in PD would improve symptomatic therapy and could provide an intervention for disease progression.

Deep Brain Stimulation (DBS) is an effective neuromodulatory therapy for treating motor signs of PD by delivering pulses of electrical current to a region of the basal ganglia (BG) via invasive electrodes. Despite not being optimized for sleep physiology, DBS slightly improves overall sleep quality in PwP by various mechanisms, including improved mobility and potential attenuation of overnight beta bursts^16–19^. Currently, DBS programming is primarily targeted towards daytime motor symptoms including tremor, stiffness, and slowness^20^. However, conventional (constant) DBS programs cannot dynamically adjust to underlying physiological states, and therefore remain at constant settings across the entire circadian rhythm, including sleep cycles. It may be that alternative stimulation paradigms are better suited for sleep physiology^21^. Thus, stimulation programs responsive to individual sleep stages may further improve therapeutic effects, such as amelioration of NREM sleep disruption and enhancement of slow wave activity. Additionally, sampling different stimulation parameters for specific sleep stages may provide a mechanistic probe on the impact of DBS to sleep physiology.

Adaptive deep brain stimulation (aDBS) allows for real-time stimulation parameter adjustment in response to brain physiology through programmed control policies and offers a means to address the aforementioned putative benefits of sleep stage-specific DBS. However, to date, aDBS efforts have targeted daytime motor symptoms and fluctuations^22–25^. aDBS provides a promising platform for sleep-specific DBS therapy because sleep provides consistent and well-characterized physiological input features for aDBS control policies. A proof-of-principle aDBS study using cortical decoding to target N3 sleep demonstrated potential to impact underlying physiology^21^.

Previous studies have demonstrated intracranial sleep stage classification; however, these were in the absence of chronic stimulation^26–31^. To advance sleep specific aDBS, it is necessary to determine the limits of sleep stage classification accuracy from intracranial signals in the presence of stimulation. Current aDBS control policies rely on simple linear classification using spectral features from intracranial data, primarily basal ganglia field potentials^32,33^. In light of the limited capabilities of aDBS embeddable classification schemes, it is necessary to validate the performance of aDBS on determining sleep stages with both constrained (e.g., for current aDBS systems) and unconstrained (e.g., for future aDBS systems) classification pipelines.

Additionally, sleep stage discrimination is typically performed on cortical data, while deep brain stimulation devices are conventionally limited to basal ganglia data. A direct comparison between the two brain regions assesses potential feasibility of sleep specific aDBS and informs future development efforts. Furthermore, a practical constraint to adoption of sleep specific aDBS is the necessity of extracranial encephalography (EEG) for conventional sleep labeling. To collect appropriate training data would therefore require multiple nights of wearing polysomnography (PSG) equipment for patients, which is an undesirable outcome. A solution to this might be personalized unsupervised approaches, e.g., direct clustering of the intracranial data for inferring groupings of sleep physiology features that may correspond to canonical sleep stages^34^. Unsupervised sleep label generation would remove the necessity for additional hardware for patients, and arguably, might better identify personalized physiology characteristics salient for neuromodulatory therapy than canonical sleep stages.

To address these needs, we developed an at-home intracranial recording and sleep stage label procurement system. This setup supports multi-night recordings of intracranial sleep data while conferring improved participant comfort compared to an in-hospital sleep laboratory. We compare personalized machine learning techniques for classifying sleep stages from intracranial neural data while participants received chronic, therapeutic deep brain stimulation. Sleep stage labels were acquired using extracranial polysomnography. We report high classification accuracy from cortical and basal ganglia data streams in the presence of chronic stimulation, and note spectral data processing techniques achieve highest accuracy. We also benchmark constrained model development for NREM classification to match compatibility with currently available sensing-enabled DBS hardware. Lastly, we demonstrate that unsupervised machine learning for NREM sleep identification presents a promising approach to personalized aDBS development while removing the need for formal sleep stage labels scored from individual polysomnography.

## Results

### Participant demography and study protocol

**Fig. 1:**
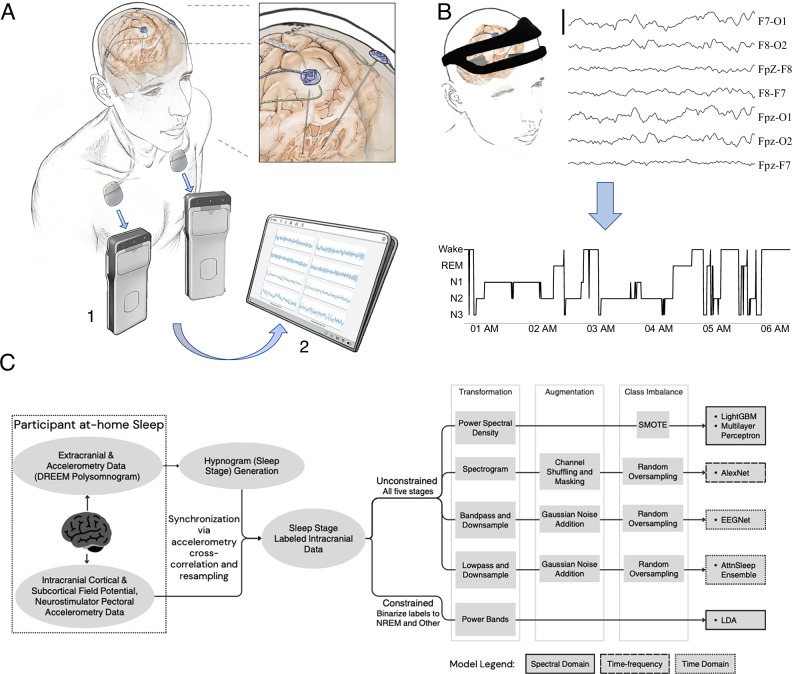
Data Acquisition and Pipeline. **A** Intracranial data acquisition: the RC + S devices concomitantly record field potentials from the basal ganglia and the cortex (inset). Data are transmitted via telemetry to relay devices (1), then to a nearby tablet (2) using bluetooth connection, and uploaded to the cloud. **B** Extracranial data acquisition: the portable Dreem polysomnogram headset was worn by the participant (top left). Dreem headband contains five dry electrodes, corresponding to seven electrode configurations. Ten-second raw EEG trace from Dreem headband shown on the right, scale bar indicates 100 µV. Accelerometry stream not shown. After the polysomnogram data stream is completed, a hypnogram (bottom) of the participant’s sleep is generated. **C** Flowchart of the complete data acquisition and processing pipeline. Data is collected via dual streams and synchronized. Labeled data is then analyzed via different data processing and model training pipelines. Top branch indicates the “unconstrained” pipeline, where contemporary approaches are benchmarked on the ability to classify all 5 sleep stages. The bottom branch indicates the ‘constrained’ pipeline, where data processing and model choices are limited to device embeddable methodologies.

Five participants were enrolled into the study. Four participants were diagnosed with idiopathic Parkinson’s disease, and one participant was diagnosed with Cervical Dystonia (Table 1). Participants were implanted with bilateral sensing and stimulation-capable quadripolar electrodes in either the Subthalamic Nucleus (STN; *n* = 2, Medtronic model 3389 lead) or the Globus Pallidus (GP; *n* = 3, Medtronic model 3387 lead). Implant site was determined by standard clinical criteria^35^. Additionally, bilateral subdural quadripolar sensing electrocorticogram (ECoG) arrays were implanted to span the central sulcus (Medtronic Resume II, model 0913025). Electrodes are connected to investigational Summit RC + S (Medtronic model B35300R) implantable neural stimulators (INS), described extensively in our previous studies^18,21,23,36^. The Summit RC + S system is capable of recording field potentials from both the cortical and basal ganglia electrode locations during chronic stimulation, and can stream data recordings at-home to a nearby tablet for offline analysis (Fig. 1A). An example of stimulation artifact in the field potential recordings, for PD03L, is provided in Supplementary Fig. 1.

**Table 1:** Patient info C-Ldopa: carboxy-levodopa

Participant ID	PD02	PD03	PD07	PD09	Dys16	Group
Age	58	66	40	48	65	Demography
Gender	M	M	M	M	M	Demography
Diagnosis	PD	PD	PD	PD	Dystonia	Demography
Duration (Years)	11	13	9	13	30	Demography
Stim target	STN	GP	STN	GP	GP	DBS stimulation settings
Pulse width (µs)	60	60	60/90	90	60	DBS stimulation settings
Stim amp. (mA)	L: 2.4 R: 3.1	L: 3.7 R: 2.8	L: 1.7–3.4 R: 1.7–3.4	L: 3–3.7 R: 3–3.7	L: 4.5 R: 3.5	DBS stimulation settings
Stim freq. (Hz)	130.2	178.6	130.2	150.6	130.2	DBS stimulation settings
Stim contact	L: C + 2– R: C + 1–	L: C + 1– R: C + 1–	L: C + 2– R: C + 2–	L: C + 2– R: C + 2–	L: C + 1– R: C + 2–	DBS stimulation settings
Medication details	C-Ldopa 25–100 mg IR (3 times daily) Aman-H 100 mg (3 times daily)	C-Ldopa 25–100 mg CR (1–2 tabs at bedtime) and 25–100 mg IR (3 times daily)	C-Ldopa 25–100 mg (1 time daily) Rasagiline (Azilect) 1 mg (1 time daily)	Rytary 195 mg (3 times daily)	–	Symptoms and clinical characteristics
UPDRS-III (OFF)	49	66	41	39	–	Symptoms and clinical characteristics
UPDRS-III (ON)	5	24	14	16	–	Symptoms and clinical characteristics
BFMDRS Movement Scale	-	-	-	-	15	Symptoms and clinical characteristics
UPDRS 1.7	No sleep symptoms	Slight sleep symptoms	Slight sleep symptoms	Mild sleep symptoms	–	Symptoms and clinical characteristics
UPDRS 1.8	No daytime sleepiness	Mild daytime sleepiness	Moderate daytime sleepiness	Mild daytime sleepiness	–	Symptoms and clinical characteristics
Sleep diagnosis	No sleep conditions	Nocturia; RBD	Daytime sleepiness	OSA; Insomnia	Restless Leg Syndrome	Symptoms and clinical characteristics
Neuropsych report (pre-op)	No reported sleep disorder or conditions	Mild sleep difficulties with nocturia and RBD	Day time sleepiness	Had long-term difficulties sleeping before PD.	Good sleep. No movements /dystonia at night. Restless Leg Syndrome at night.	Symptoms and clinical characteristics
Left device	60.3	68.4	42.9	47.1	57.2	Total Hours Recorded
Right device	57.0	45.3	48.0	41.3	45.5	Total Hours Recorded

*Aman-H* amantadine-hydrochloride, *RBD* REM-sleep behavior disorder, *OSA* obstructive sleep apnea, *UPDRS* unified Parkinson’s disease rating scale (obtained prior to DBS implantation).

### Chronic recording of intracranial activities

Classifying sleep stages from intracranial data requires synchronizing the intracranial data with sleep stage labels collected from an extracranial polysomnogram (Fig. 1A, B). Participants therefore wore a portable, polysomnogram headband (Dreem 2 headband, Beacon Biosignals Co.) while simultaneously recording field potentials from the RC + S devices during at-home sleep over multiple nights^21,37^. The Dreem wearable headband provides an automated hypnogram (i.e., sleep stage label for each 30 s epoch) generated from electroencephalography (EEG) and additional (pulse oximetry, electromyogram, and accelerometry) data streams. The hypnogram provides sleep stage labels comparable in accuracy to a conventional, manual scored PSG^37^. Participant data acquisition occurred on mostly consecutive nights, with some intermittent breaks when requested by the participants. Most participants completed the entire data collection in approximately two weeks.

Three intracranial field potential (FP) streams were sampled in millivolts at a rate of 500 Hz. The basal ganglia local FP was sampled using a bipolar ‘sandwich’ electrode configuration, where the resultant data stream is the electric potential difference between the two electrodes immediately adjacent to, and therefore equidistant to, the electrode delivering stimulation. Cortical FPs were acquired in two data streams: a bipolar configuration on the precentral gyrus, and a wide bipolar configuration spanning the central sulcus. Each participant recorded ~ ten nights of external polysomnogram and intracranial field potential recordings during chronic therapeutic stimulation.

We next aligned the sleep stage labels from the polysomnogram headband with the intracranial FP (Fig. 1C). Participants were asked to simultaneously manually perturb the DREEM headband and their INSs to introduce intentional, synchronized artifacts in the respective accelerometry streams. This was used as a first line synchronization method. To overcome potential sampling rate discrepancies and temporal offsets between the Dreem and RC + S data, we resampled the extracranial streams. Secondly, cross-correlation analysis was applied to the whole night accelerometry streams of each recording device to validate the manual synchronization and identify potential lags or offsets in the clock rates. If lags or offsets were detected, the extracranial stream was temporally re-aligned to maximize cross-correlation with the intracranial accelerometry, thereby aligning the intentional artifacts. (see Anjum et al.^18^ for more details).

The resultant dataset consists of a sleep stage label (e.g., N1, N2, N3, REM, Wake) for each 30-second epoch of the three intracranial data streams (basal ganglia, precentral gyrus, cross-central sulcus) for each hemisphere.

### Spectral distributions show sleep-specific changes of sleep stages

**Fig. 2:**
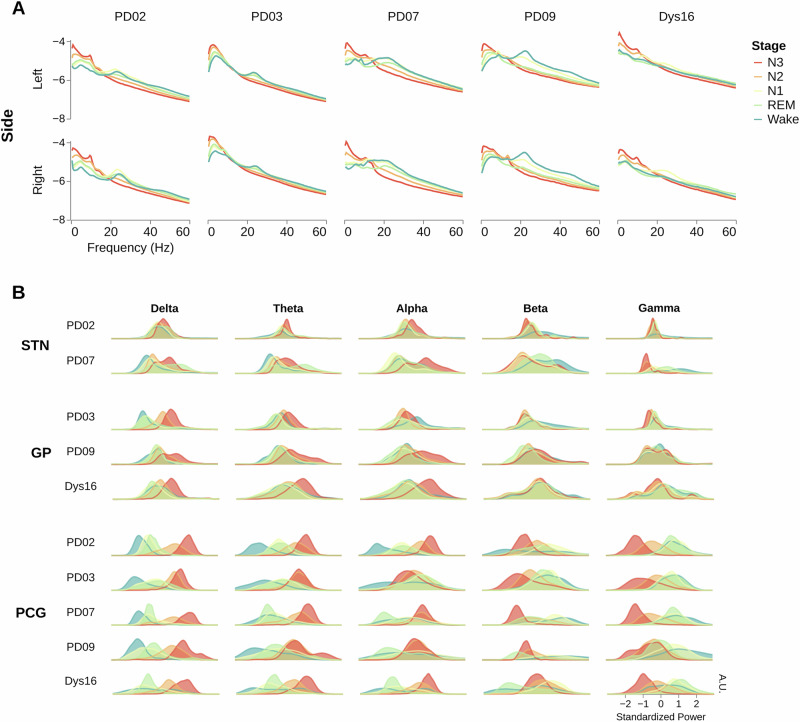
Electrophysiology Across Sleep Stages. **A** Power spectral density plots from the precentral gyrus for each participant, partitioned by sleep stage (legend). *Y*-axis corresponds to the log base 10 of the spectral power. Top row depicts left device, bottom row depicts right device. **B** Log-standardized distributions of canonical power bands colored by sleep stage, with area for each color renormalized to one. Legend shared with panel A. Left and right hemispheres were aggregated together, prior to standardizing. Each row is a participant, with row groupings indicating a brain region. Columns indicate frequency bands in which frequency powers were summed. STN: Subthalamic Nucleus, GP: Globus Pallidus, PCG: Precentral Gyrus. Delta: 1–5 Hz, Theta: 4–8, Alpha: 8–12, Beta: 12-30, Gamma: 30–60.

Sleep stage transitions are associated with changes in canonical frequency bands on EEG data^5^. To verify that intracranial field potentials reflect the expected spectral changes across sleep stages, we analyzed the distributions of canonical intracranial power bands across sleep stages from each brain region (Fig. 2). In accordance with EEG-based profiles of sleep stages, intracranial epochs corresponding to N2 and N3 sleep showed higher delta, alpha, and theta power, and decreased gamma power. Compared to NREM sleep stages, REM sleep more closely resembles wakefulness^38^.

### Spectral preprocessing outperforms time series on sleep classification

**Fig. 3:**
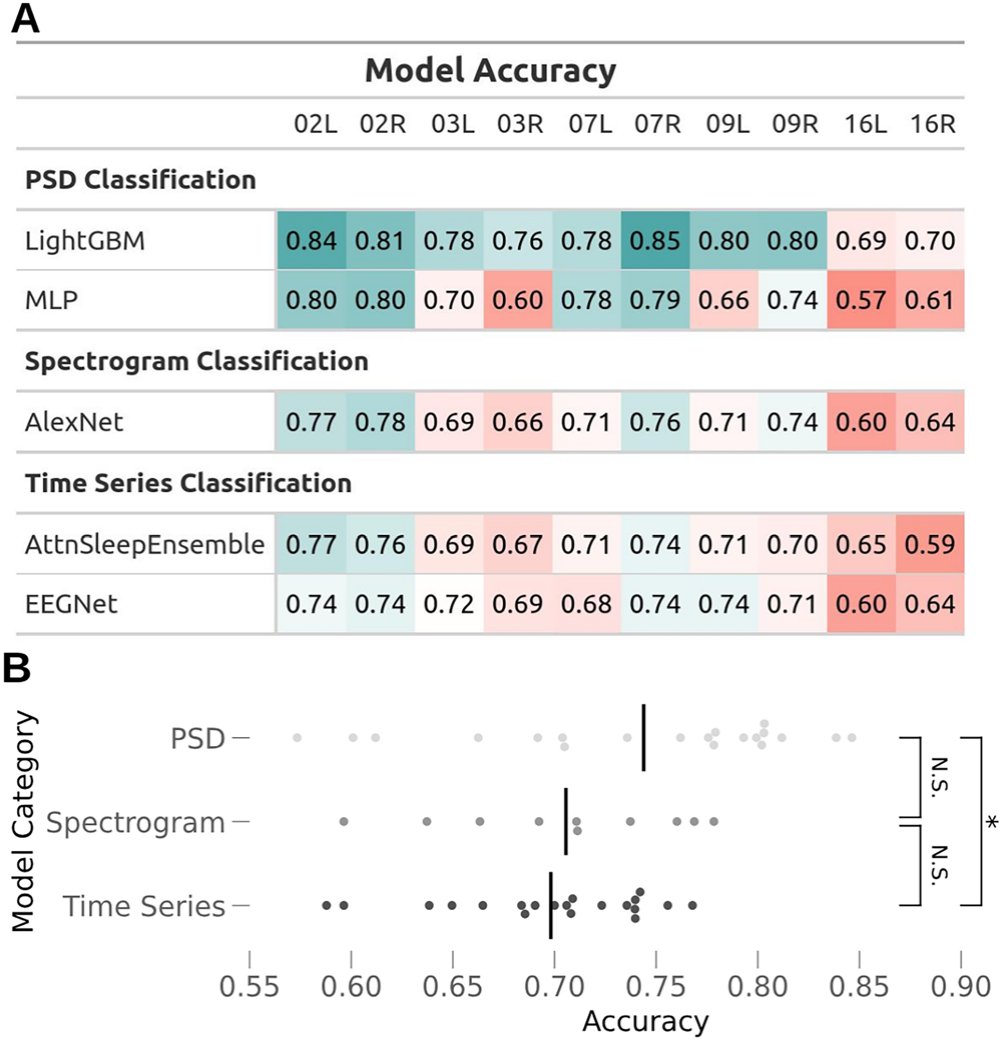
Performance of 5 stage sleep stage classification models. **A** Table showing model accuracy on hold-out test set for each hemisphere. Numbers indicate the proportion of sleep stages correctly classified. Teal indicates better performance, while red indicates worse performance. **B** Scatter plot of time domain models’ accuracy compared to that of spectral models (Linear Mixed Model, using each unique grouping of (model, hemisphere) as the random effect. PSD vs Time Series; *p* = 0.039; *n* = 20. PSD vs Spectrogram; *p* = 0.25; *n* = 20/10. Spectrogram vs Time Series; *p* = 0.36; *n* = 10/20).

We tested the performance of the five models on the sleep stage classification task (N1-3, REM, and Wake; 5 stages total) for each hemisphere, as described in 3.3.1 and 3.3.2 (Fig. 3A). Models were trained on all three available field potential streams (basal ganglia, precentral gyrus, and cross-central sulcus; e.g., BG + CTX). Model performance was assessed via accuracy, indicating the proportion of correct predictions with respect to all predictions on the stratified hold-out test set.

LightGBM correctly classified the highest proportion of sleep epochs for the five sleep stages across the PD participants, achieving 80.3% (±0.9% SEM) average accuracy. There is only a marginal reduction in accuracy to 78.5% (±1.3% SEM) when including the dystonia participant (Fig. 3A). This result holds when the models are trained without the addition of class imbalance correction and data augmented samples (Supplementary Fig. 3), and when intracranial data from across the two brain hemispheres are aggregated into a single dataset (Supplementary Fig. 5).

N1 achieved the lowest sensitivity across all models and classes (Supplementary Figs. 6, 7). This is a common result in sleep staging due to the high variability of features and relatively low occurrence of N1 sleep^37,39^. The majority of misclassifications occurred between the N2 and N3 stages (Supplementary Fig. 7). These results reflect the overlapping distributions in spectral power bands fundamental to defining N2 and N3 sleep, particularly delta (Fig. 2B)^40^. N2 and N3 are traditionally differentiated via the prominence of slow wave activity, and the occurrence of K-complexes and sleep spindles in N2 and their absence N3^5^. These transient electrophysiological activities may not be captured by the spectral preprocessing, nor fully identified by currently available time series models.

Sleep classification performance was highly dependent on model selection and preprocessing. Specifically, the models trained on PSD vectors, i.e., the spectral models, had significantly higher accuracy than models trained on time series (*p* = 0.039), exhibiting an average accuracy increase of 5.1% (Fig. 3B). Models trained on PSD vectors also exhibited higher accuracy than AlexNet trained on spectrograms, but did not reach statistical significance (*p* = 0.25). There was no substantial statistical trend between AlexNet and models trained on time series data (*p* = 0.36).

### Feature importance for sleep-stage detection

LightGBM, the highest performing model, also allows for interpretation of the relative importance of features towards classification accuracy. Our LightGBM ingested PSD vectors from all three field potential streams (basal ganglia, cross-central sulcus, and precentral gyrus), with 0.5 Hz frequency bin resolution, as feature inputs. Partitioning the dataset based upon a threshold (e.g., all data points less than a specific value are separated from data points greater than the value) for a specific feature is often called “splits”^41^. The gain of a split refers to the improvement in predictive accuracy achieved when performing a particular split. For the LightGBM reported here, the gain for each frequency bin corresponds to the increase in sleep stage prediction accuracy when splitting the dataset into subsets that are smaller or greater than a determined power value for that frequency bin.

**Fig. 4:**
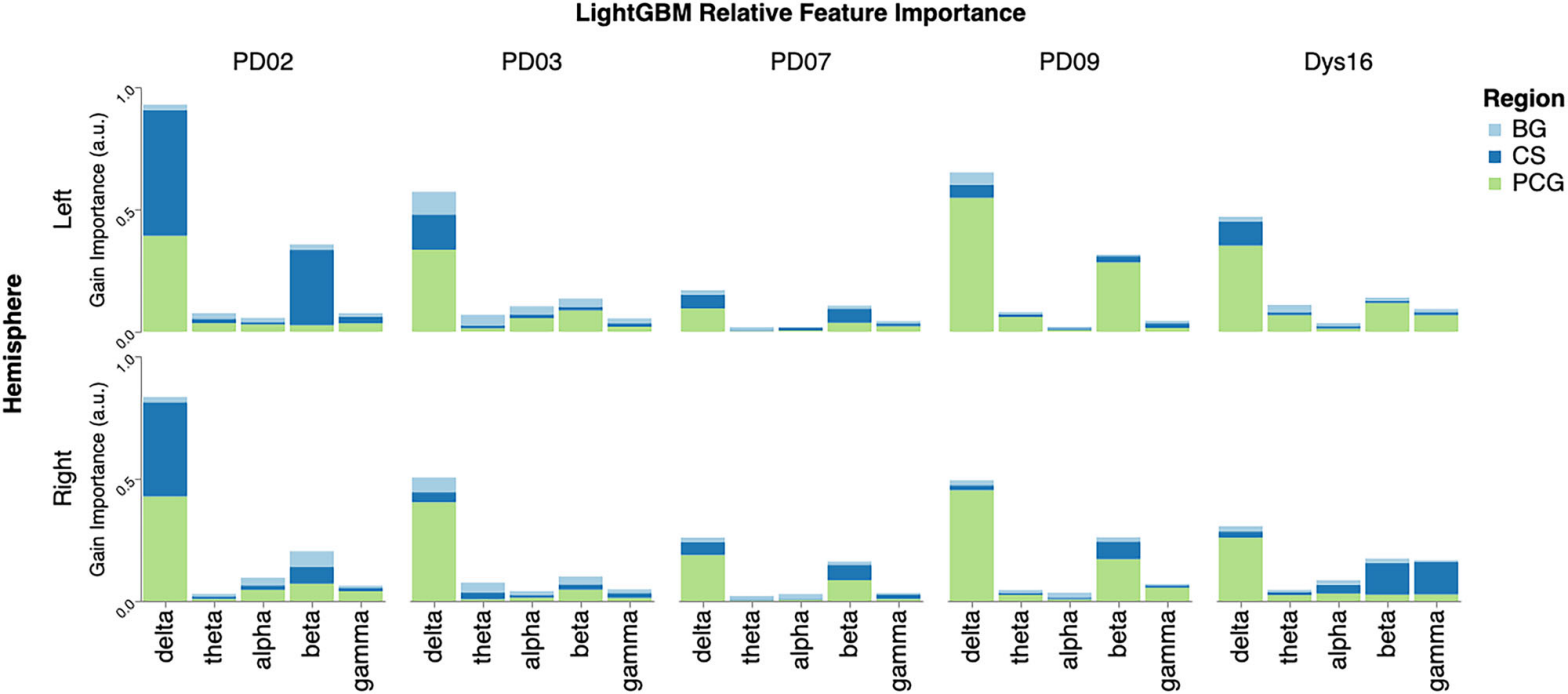
Feature importance for LightGBM. Maximum split gain within each canonical power band. Rows indicate the brain hemisphere, and columns indicate participants. Color indicates the brain region: BG basal ganglia; CS cross-central sulcus; PG precentral gyrus.

We group the frequency bins into their canonical powerbands, and report the maximum gain across splits for the frequency bins within each powerband (Fig. 4). For example, the gain reported for the basal ganglia theta band is the maximum gain across the frequency bins within the 4.5–8 Hz range.

The most informative split occurred within the delta band (0.5–4 Hz) of a cortical channel for every brain hemisphere analyzed. For most hemispheres, this split occurred from the precentral gyrus delta band. The second most informative split occurred within the beta band.

### Region-specific sleep classification

We next analyzed model performance across brain regions for two primary reasons. Firstly, we hypothesized that cortical data would confer an advantage over basal ganglia data, because cortical data is more closely aligned to our ground truth sleep labels derived from PSG (cortical) electroencephalography. Therefore, cortical data may show more distinct physiological activities across sleep stages (Fig. 2B), and basal ganglia data are more susceptible to stimulation artifacts. Secondly, analyzing sleep classification solely utilizing basal ganglia data is important for potentially generalizing sleep aDBS to standard clinical DBS lead configurations that do not include cortical sensing.

**Fig. 5:**
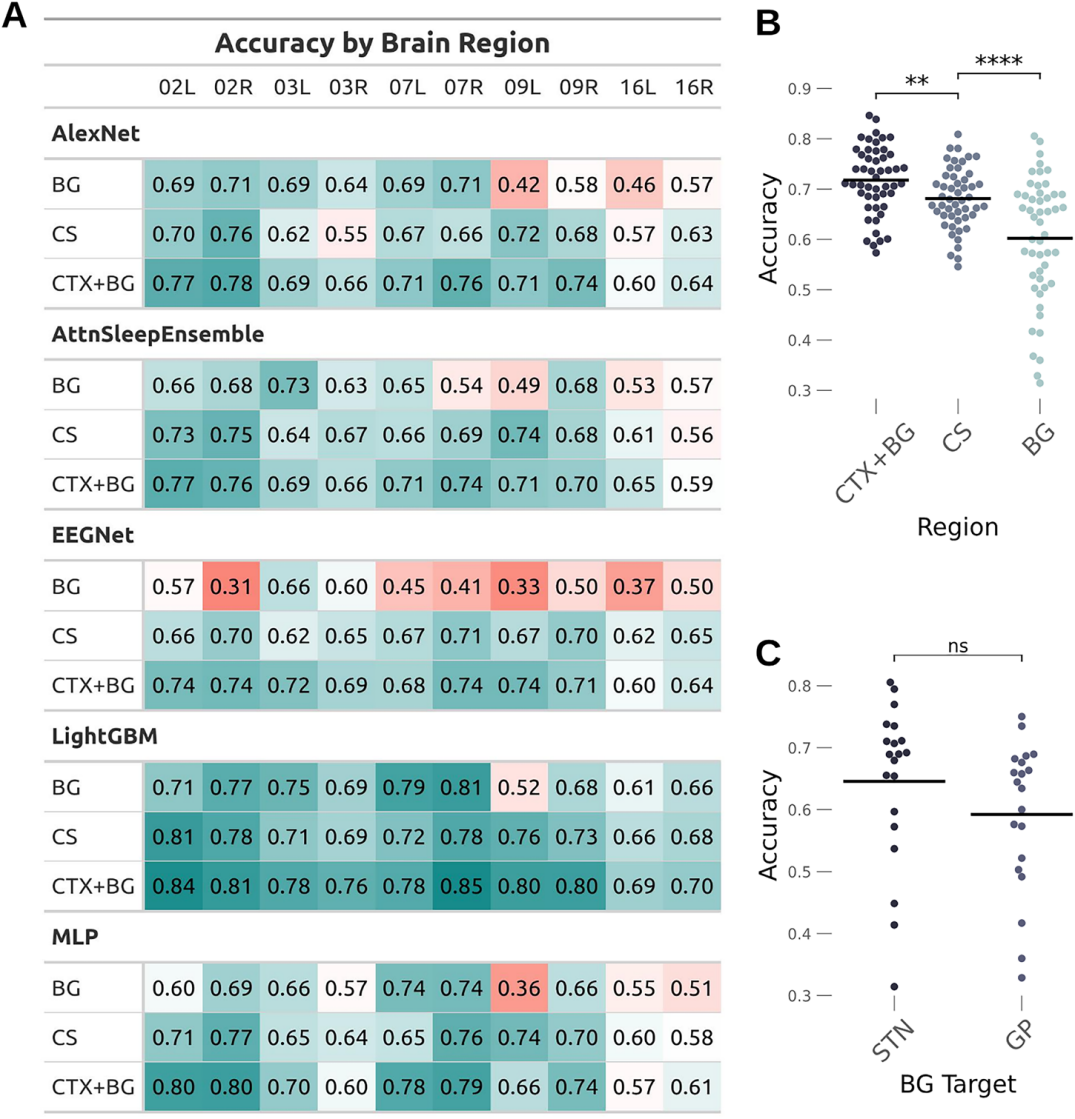
Model performance segregated by brain region. **A** Table of model accuracies on hold-out test set, partitioned by brain region field potential used for model training and testing. Teal indicates better performance, while red indicates worse performance. **B** Comparison of all models across all hemispheres when trained on Basal Ganglia (BG) field potentials versus Cortical (cross-central sulcus; CS) field potentials (Linear Mixed Model [LMM], using each unique grouping of (model, hemisphere) as the random effect; *p*«1e-4; *n* = 50) and CS vs Dual Cortical and BG (i.e. CTX + BG) (Linear Mixed Model [LMM], using each unique grouping of (model, hemisphere) as the random effect; *p* < 1e-2; *n* = 50) **C** Comparison of all models when trained on STN (participants 02,07) field potentials versus GP (participants 03,09) field potentials (LMM with (model, hemisphere) as random effect; *p* = 0.12; *n* = 20). Participant 16 was excluded in subpart C to focus this analysis on participants with Parkinson’s disease.

Therefore, each model was trained on either the basal ganglia stream (BG), the cross-central sulcus stream (CS), or the basal ganglia, precentral gyrus, and cross-central sulcus streams together (CTX + BG) (Fig. 5). Note that the CS and BG setups each consist of a single field potential stream, to draw a fair comparison between cortical versus basal ganglia FPs for classification. The CTX + BG contains all three streams to provide a ceiling on classification performance from the available data.

Consistent with the previous results, LightGBM was the top performer across CS and BG streams, with an average accuracy of 73.2% and 69.9% on 5 class sleep stage classification, for CS and BG, respectively. Interestingly, LightGBM also achieves the smallest performance discrepancy between CS and BG among all models, with an average difference of 3.3%, as compared to the next smallest discrepancy, 3.9% for AlexNet and 19.3% for EEGNet, the largest discrepancy. At the group level, model performance was significantly improved when trained on cross-central sulcus (CS) field potentials as compared to BG field potentials, as cross-central sulcus data conferred an 8.4% boost in average accuracy (Fig. 5B; Linear Mixed Model [LMM], using each unique grouping of (model, hemisphere) as the random effect; *p* < 1e-4). Furthermore, combining the cross central sulcus, precentral gyrus, and BG field potentials (CTX + BG) significantly improved accuracy compared to just the central sulcus field potential (CS) (Fig. 5B**;** Linear Mixed Model [LMM], using each unique grouping of (model, hemisphere) as the random effect; *p* < 1e-2), improving the average accuracy across all models by 3.7%. There was no significant difference in classification accuracy between subthalamic and pallidal field potentials (Fig. 5C), indicating both regions are potentially viable targets for subcortical intracranial sleep staging in this small sample. Additionally, model accuracies across all regions are comparable when assessing performance on a hold-out test set of two complete, randomly chosen nights, which were not used in training (Supplementary Fig. 4).

### Performance of embeddable models

The previously described machine learning models and preprocessing techniques provided a useful benchmark for the sleep classification performance achievable with intracranial data. However, the features and models cannot be implemented in currently available, sensing-enabled neurostimulation due to hardware constraints. To assess the capability of classifications schemes constrained for implementation on currently available DBS hardware, we next analyzed the performance of linear models on discriminating NREM (N1-3) from other stages (REM and awake). We include results for LightGBM and the entire PSD vector to draw an explicit comparison between embeddable, constrained methodologies and the unconstrained methodologies above.

**Fig. 6:**
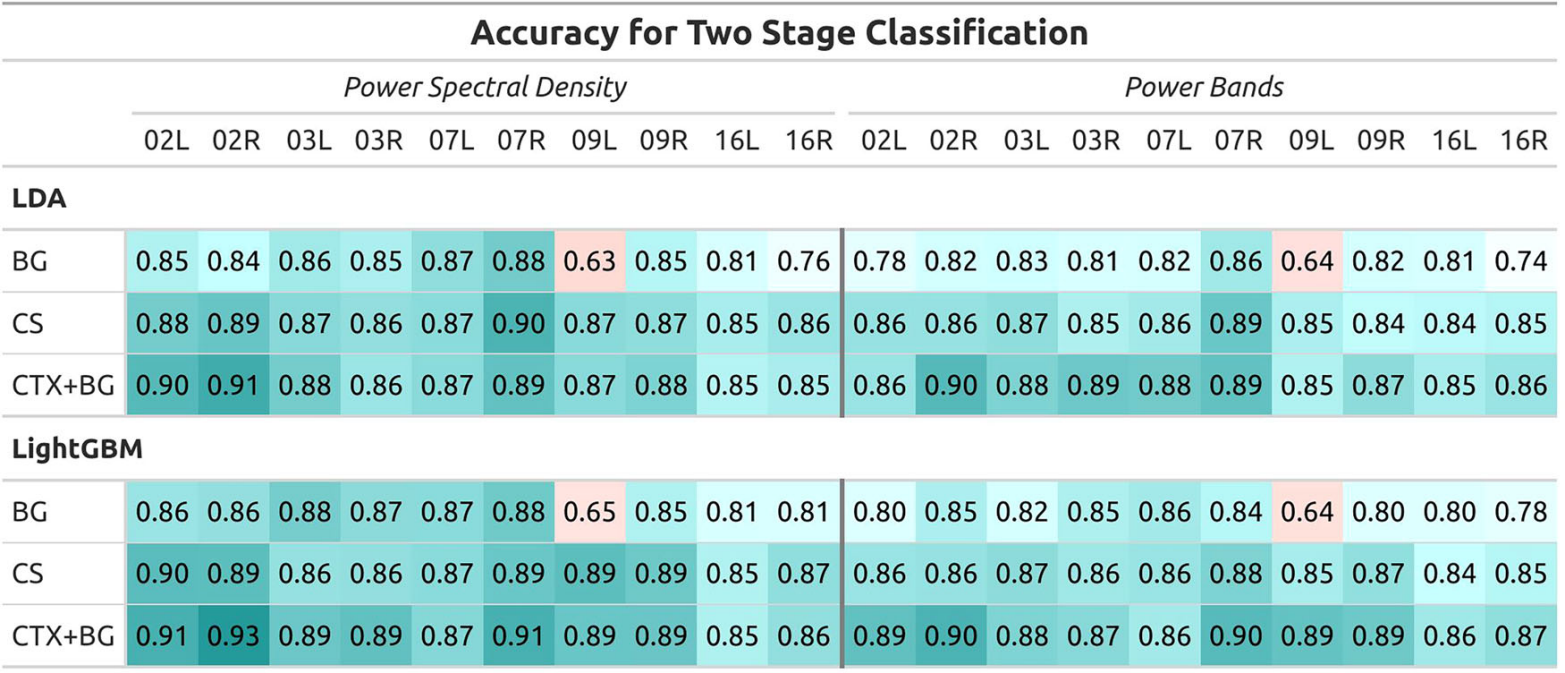
Two-stage classification performance for embeddable models. Accuracy for NREM vs Other (REM and Wake) classification on hold-out test set.

The average 2-stage NREM classification accuracies for LDAs trained on BG, CS, and CTX + BG power band data are 79.4% (±1.2% sem), 85.6% (±0.4%), 87.3% (±0.5%), respectively (Fig. 6. This is only a marginal decrease from the average accuracy that was found when the entire power spectral density vector was used for training the same models (81.9%, 87.2%, 87.5%, respectively). Linear Mixed Model regression of accuracy comparing power band versus PSD classification models (using hemisphere as a random effect) did not show significance, for any brain region (*p* = 0.21, 0.13, 0.41, respectively). LDA performance without the log transform preprocessing step yielded similar results, as did testing model performance on a hold-out test set of two nights (Supplementary Figs. 8 and 4, respectively). Additionally, the performance improvement when using the more powerful LightGBM (versus LDA) is small, with the average difference between LightGBM and LDA on the canonical power bands for BG, CS, and CTX + BG as 1%, 0.3%, and 0.6%, respectively.

### Unsupervised clusters as sleep stage label surrogates

**Fig. 7:**
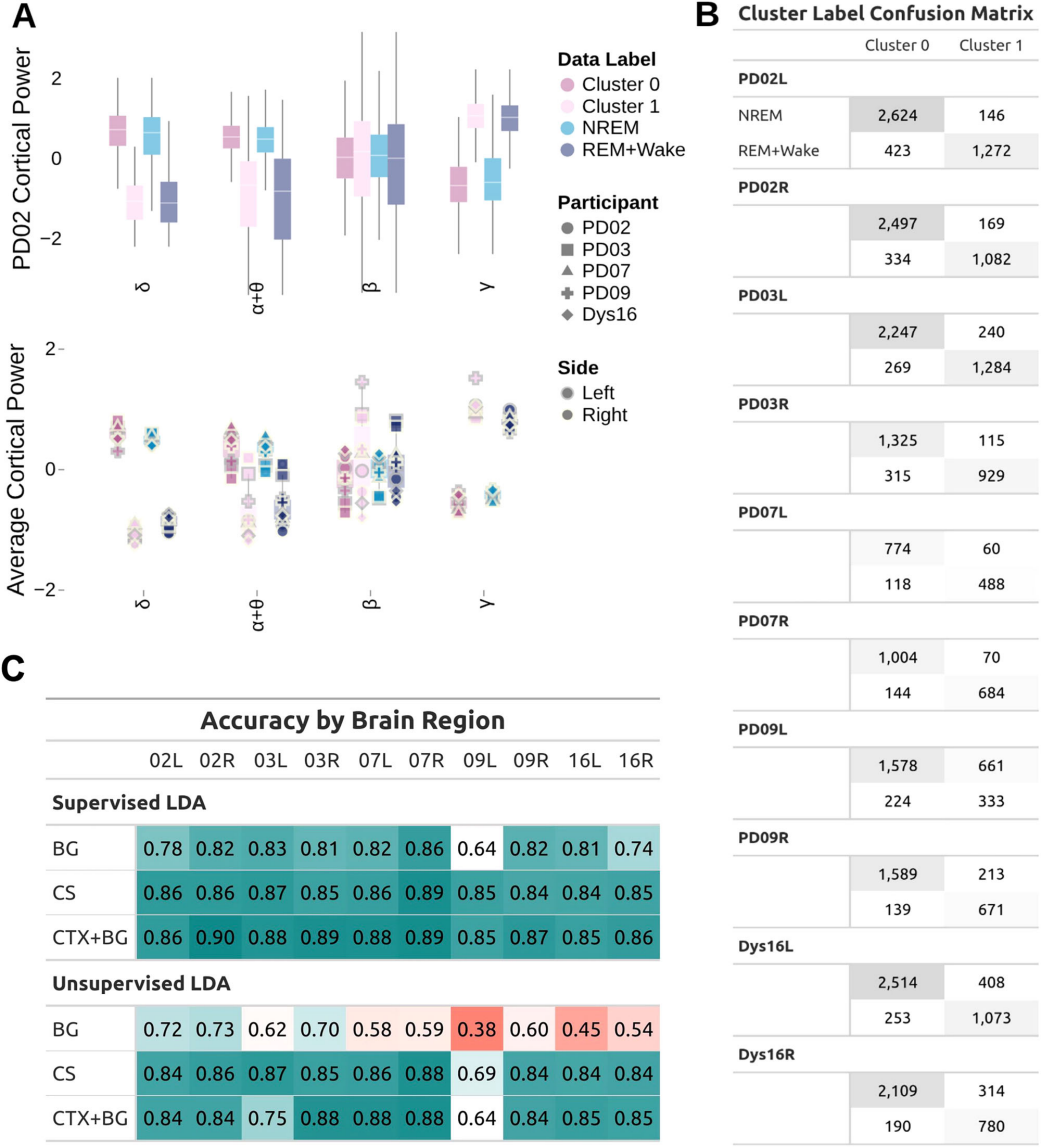
Unsupervised Learning of NREM sleep. **A** Comparing distributions of features in the training set for the supervised vs unsupervised approach, from precentral gyrus data stream. Top - Example participant (PD02 Left device). Each column grouping consists of box plots summarizing the distributions of that particular power band. Colors indicate the classification label used for training the LDA. Bottom - The average of each power band for each participant, partitioned by classification label. Points depict each participant’s average power. Box plots represent distribution of average power across participants. **B** Table depicting the confusion matrix between surrogate class labels and true sleep stage labels for each participant’s training set. Numbers indicate the total number of data points that belong in the intersection between the label conditions. **C** Table showing the accuracy of LDA trained with canonical power bands and sleep stage labels (Supervised LDA) vs cluster labels (Unsupervised LDA) on a hold-out test set of sleep stage labeled power bands, partitioned by brain region.

**Fig. 8:**
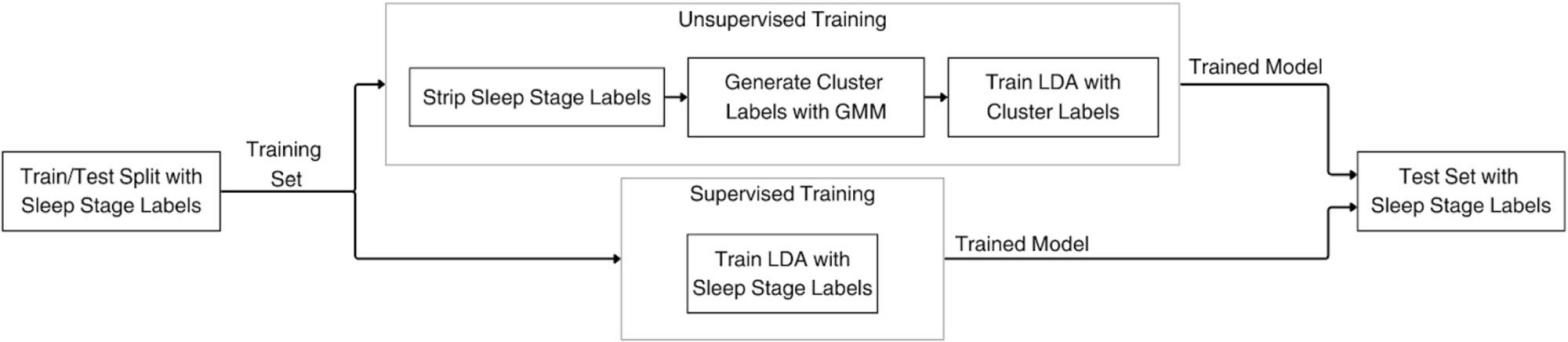
Flowchart of unsupervised vs supervised classification of NREM sleep. The bottom pathway conveys the conventional supervised learning approach. Additionally_,_ the (top) unsupervised pathway generates surrogate cluster labels using GMMs. An LDA is trained on the cluster labels, but tested on the same hold-out test set containing sleep stage labels as the supervised learning approach to ascertain how closely the unsupervised labels match the supervised labels.

Lastly, we explore the efficacy of individualized unsupervised (generated) clusters, generated using Gaussian Mixture Modeling, as surrogates for NREM vs other sleep stage labels (see Fig. 8 for label generation pipeline). Across all participants and hemispheres, a cluster on the powerband data was generated that closely resembled the NREM sleep (Fig. 7A). This cluster corresponded to a grouping of the data points with high delta power and low gamma power, which matches the spectral properties of NREM sleep (Supplementary Fig. 9). The distribution of true class labels for each cluster across participants is shown in Fig. 7B. Notably, the average accuracy decrease between the “unsupervised” vs “supervised” approaches, i.e., when the LDA models were trained using the true labels or unsupervised derived surrogates, for predicting NREM stages on the test set was 20.8%, 1.9%, and 4.6% for basal ganglia, cross-central sulcus, and basal ganglia plus cortical streams, respectively, indicating that unsupervised approaches may reasonably recapitulate NREM labels (Fig. 7C). These results were corroborated when assessed on a hold-out test set of two nights (Supplementary Fig. 4).

## Discussion

This study employed a novel, at-home data acquisition set-up using investigational, bidirectional neural interfaces to systematically explore intracranial sleep stage classification during chronic deep brain stimulation. We demonstrated that the five sleep stages can be classified from intracranial field potentials with ~80% accuracy using supervised machine learning methods. Basal ganglia field potentials alone can achieve over 75% accuracy in discriminating sleep stages, but the addition of cortical electrocorticography confers additional accuracy. We also found that unsupervised machine learning methods can closely recapitulate NREM sleep labels for simplified NREM classification paradigms. Linear models that can be programmed into currently available DBS devices achieved an average accuracy of 85% for PD participants when using cortical power bands. There are multiple potential machine learning options for classifying sleep stages from intracranial data for personalized sleep staging models. The raw time series, or lightly processed time series, can be passed into deep artificial neural networks with architectures designed for extracting periodic relationships from data. Alternatively, the data can be transformed into the spectral domain and fed into simpler models, such as gradient boosting machines, that operate on lower-dimensional inputs. We chose to compare both: a gradient boosting machine method (LightGBM) and a selection of deep neural networks, where the deep neural networks span a range of architectures specifically directed towards the sleep staging task^42^.

LightGBM trained on spectral features outperformed time-series based deep neural networks on intracranial sleep stage classification. As sleep stages are largely defined from the neural oscillatory activity at band frequencies ranging from delta to beta range (1–30 Hz) in sleep scoring manuals (i.e., the gold standard), models that directly learn from spectral features may have an advantage, particularly for more limited datasets^5^. Hyperparameter tuning is also computationally cheaper and faster for LightGBM than deep models^43^.

The finding that gradient boosting methods outperform deep neural networks on a complex supervised classification task is not unique to this study. Borisov et al. demonstrated that gradient boosting methods outperform deep neural networks on small to medium sized tabular datasets^44^. In a comparative study of machine learning techniques applied to sleep classification, Sekkal et al. report that decision tree based methods (similar to gradient boosting machines) perform comparably to neural network approaches^45^. Furthermore, Yin et al. demonstrated that LightGBM can be an effective, general model for cross-subject sleep classification from basal ganglia field potentials in the absence of stimulation^29^.

In this study, despite the unique multi-night per subject recordings, there may not be enough data samples for deep models to eclipse the performance of gradient boosting models. The time-series models do not benefit from extensive spectral preprocessing of the data, and therefore must implicitly learn periodic and cross-frequency relationships from high variance time series vectors, or bandpassed vectors, during training. Increasing the number of nights recorded may improve deep neural network performance relative to LightGBM, but may not be practically feasible or sufficiently add value in most scenarios. Additionally, AttnSleep and EEGNet were originally designed for extracranial field potentials, while we apply them to intracranial field potentials. Differences in signal characteristics between extracranial and intracranial field potentials may contribute to the relatively lower sleep classification accuracy reported here.

This study employed personalized models for sleep stage classification, as we do not report a general sleep classification model effective on field potentials during chronic stimulation. Our motivation for developing models at the personal level is twofold. Firstly, we aim to identify an upper limit on intracranial sleep classification and a resultant comparative analysis of sleep classification across brain regions. Secondly, we explore the efficacy of unsupervised learning approaches for identifying personalized sleep stages that correspond to established, traditional sleep stages.

Previous high quality studies have demonstrated sleep stage classification from basal ganglia activity recorded from DBS electrodes^26–31^. However, these endeavors withheld therapeutic stimulation because stimulation tends to introduce recording noise and alter electrophysiological biomarkers potentially important to classification^46^. Chronic stimulation raises concerns for intracranial sleep stage classification due to potential noise introduced into field potential recordings and modulation of neural activity by DBS, which may alter key oscillatory biomarkers for sleep stage classification^46,47^.

To our knowledge, this is the first report of sleep stage classification from intracranial field potentials while participants receive chronic stimulation. Although we demonstrate that constant DBS generally does not greatly disrupt sleep stage discrimination, this may not definitively be the case with adaptive DBS, during which the stimulation amplitude (or other parameters) changes. Therefore, future studies should investigate whether changes in stimulation, such as during adaptive DBS, introduce noise or spectral distortions that may further disrupt sleep stage discrimination. These two potential issues might be alleviated by the inclusion of cortical data, which are less susceptible to DBS-related artifacts.

We note that sleep stage classification is significantly improved when using cortical field potentials over basal ganglia potentials, but do not observe a significant difference between subthalamic and globus pallidus performance. Given that sleep stage labels are largely determined from scalp EEG data for current scoring standards, the superior performance of cortical data is likely a reflection of the cortical field potentials having greater fidelity to the scalp EEG than the basal ganglia data, as well as differences in signal-to-noise. The performance improvement of cortical over BG field potentials will likely widen during aDBS, as changing stimulation amplitude (or other parameters) introduces additional artifacts into the field potential recordings. Including both cortical streams and the basal ganglia stream would likely ensure better classification accuracy.

Notably, we report classifier performances on BG field potentials that are similar to studies conducted with stimulation OFF^26,28^. Therefore, not only do the STN and GP display discriminable physiological activity across sleep stages, but these sleep-stage specific neurophysiologies are detectable by suitably chosen models during chronic stimulation^48^. The greater variance in classifier scores on BG field potentials versus cortical field potentials is plausibly due to increased noise introduced by chronic stimulation. Across all brain regions, delta power was the most informative feature for classifying sleep stages, which may be used as a primary feature for linear models that necessitate simpler inputs.

We note that classifier performance was consistently lowest for the participant diagnosed with cervical dystonia versus Parkinson’s disease, even when using individualized model training. Therefore, it is plausible that the pathophysiological markers specific to a neurodegenerative disorder may affect the quality of intracranial sleep classification. Indeed, although the spectral profiles of NREM sleep stages for this dystonia patient were generally consistent with the PD participants, there is greater variance in the “Wake” stage for the low frequency power bands (Fig. 3B). Increased power in the theta band (dystonic tremor frequency) is a hallmark physiomarker of dystonia, and may have contributed to reduced sensitivity of “Wake” sleep stages compared to PD participants (Supplementary Fig. 6)^49^.

Additionally, PD03 had a notable absence of power in the 11–15 Hz band during NREM sleep (Fig. 3A). As PD03 also had the highest UPDRS scores (Table 1), it is possible that this participant’s more advanced Parkinson’s disease led to greater attenuation of spindle activity. Reduction in the 11–15 Hz band due to PD related sleep denaturing, potentially in core physiological features defining N2 sleep, may plausibly explain the reduction in N2 classification specificity for PD03 (Supplementary Fig. 6). Similar pathophysiological confounds as those for PD03 and Dys16 may arise in other neuropsychiatric or neurological disorders, and may warrant alternative methods, or unsupervised methods, of sleep staging to account for these differences.

Current Deep Brain Stimulators are limited to power bands as features for linear models, and cannot employ the aforementioned (unconstrained) classification pipelines. Therefore, we assessed the performance of linear models on discriminating NREM from combined REM and Wake using power band data outlined in a “constrained” pipeline. Within this simplified paradigm, the majority of Linear Discriminant Models achieved performance of over 80% accuracy using basal ganglia data alone. NREM classification using linear models can be implemented using commercially available devices (e.g., Percept), providing a potentially accessible approach for simplified sleep-aware DBS^32^.

Supervised sleep classification requires adequate sleep stage labels for model training. However, obtaining sleep stage labels necessitates external hardware for polysomnogram data acquisition and specialized software, or a trained neurologist, for interpreting the data. These dependencies limit the scalability of intracranial sleep classification for individuals with DBS using supervised methods. Additionally, sleep stages are a historical discretization of the complex dynamics observed during sleep based on the available extracranial (electroencephalography) methods at the time, and may overlook individualized nuances in neurophysiology that occur within and across stages. Unsupervised learning is a category of machine learning that identifies patterns within data without the explicit use of external labels^41^. Therefore, due to the consistent spectral properties of traditional sleep stages (Fig. 2), unsupervised learning approaches may adequately approximate sleep stage labels. Furthermore, unsupervised methods also have the potential to uncover granular, personalized physiological dynamics that do not fit neatly into classic sleep stages, such as bursts of beta activity^18^.

In an effort to circumvent the need for externally acquiring sleep stage labels, we explored unsupervised methods for generating surrogate NREM labels from power band data. Notably, unsupervised methods clustered the data into groups closely resembling the NREM versus REM/Wake sleep stages. Particularly, after light data filtration, Gaussian Mixture Models correctly identified a data cluster with high delta power and low gamma power across all participants and hemispheres (Fig. 5A). LDA models trained on these surrogate labels performed comparably to models trained on the real sleep stage labels, directly supporting a close correspondence.

Unsupervised approaches to sleep classification circumvent the need for burdensome headgear for label generation. Additionally, unsupervised methods may leverage variability in sleep physiology across individuals, as new sleep labels are identified for each individual’s specific sleep physiology. This may be of particular importance to individuals with neurodegenerative disorders. The neurodegenerative disorder may disrupt sleep physiology for an individual. For example, PwP tend to show reduced slow waves, therefore reducing the applicability of conventional sleep staging^6^. Consequently, classifying N3 sleep by analyzing absolute delta power may not be appropriate for PwP. Unsupervised (personalized) methods would account for this change in neurophysiology by analyzing relative changes of delta power for an individual. As neurodegenerative diseases progress, unsupervised methods would be a simple and fast method for regular updating of aDBS control policies.

There are some limitations to the present study. We utilized sleep stage labels collected from a portable polysomnogram (PSG) and proprietary sleep staging algorithm as ground-truth^37^. The PSG and corresponding sleep staging algorithm (DREEM headband) were recently validated in an aging population by Ravindran et al.^50^. Ravindran et al. demonstrated that DREEM automated sleep staging has suitable concordance with standard PSG, particularly for deep NREM, although N1 and REM staging classification was slightly reduced. Portable PSG is a departure from the gold-standard of sleep stage labeling by a certified neurologist in an in-hospital sleep laboratory. Indeed, we note that most misclassifications on the five sleep stage discrimination problem occur between N2 and N3 sleep. This is reflected in Ravindran et al., where the majority of N3 misclassifications are also N2 predictions for PSG-determined sleep stage labels (as compared to sleep scoring by certified neurologists). However, we report overall high accuracy, 80% for PD participants, for five stage sleep classification. Additionally, using portable PSG permitted multi-night naturalistic (at home) data collection, and intracranial field potentials demonstrated expected changes in the canonical power bands across the PSG generated sleep stages. The fully automated data acquisition and labeling system developed here confers the benefits of patient comfort, multi-night recordings, and general scalability, while the classical manual sleep scoring approach is often resource-limited.

Our results highlight the advantages of cortical field potentials for sleep stage discrimination. However, surgical implantation of chronic ECoG strips is invasive, and commercially available DBS systems are not designed for cortical recording. Therefore, the benefits of intracranial cortical data are currently not accessible at scale. The inclusion of low-profile, wearable devices that collect extracranial cortical data, or other relevant biometrics, could act as surrogates for intracranial cortical data. These alternative data streams could be temporally synchronized with basal ganglia field potentials and putatively improve classification accuracy.

Furthermore, we report a low subject number (*n* = 5). Validating that the findings reported here apply to a larger cohort will require future efforts. Nonetheless, the large amount of data collected per individual (~50 h per participant) allows for robust within-individual model development for sleep stage classification.

In a previous study, we showed that a closed-loop control policy can adaptively modulate DBS parameters in response to real-time discrimination of N3 sleep via a device-embedded classification model^21^. We expand upon those results and demonstrate that five-stage sleep classification is possible using cortical or subcortical recordings in the presence of stimulation, with cortical data providing a boost in performance. We also demonstrate that NREM sleep can be consistently identified using supervised classification schemes currently implementable in available DBS devices. Furthermore, NREM sleep can be accurately distinguished from REM and Wake using unsupervised clustering methods even with basal ganglia recordings alone, indicating potential aDBS policy development without sleep stage labeling. Together, these findings support next steps towards implementing and scaling aDBS for sleep in PD^51^.

## Methods

All participants were enrolled as part of a parent study exploring adaptive deep-brain stimulation for daytime movement symptom fluctuations^23,24^. The Institutional Review Board (Human Research Protection Program) of University of California, San Francisco approved the parent study protocol (18-24454, 2 August 2018), and the study was registered on clinicaltrials.gov (NCT0358289; IDE G180097).

### Data preprocessing and unconstrained model training

Participant FP streams were standardized to have zero mean and a standard deviation of one for each individual night, and subsequently segmented into 30 s epochs. Each data epoch contained only a single sleep stage label, and data epochs with missing data (i.e., epochs shorter than 30 s) due to streaming disconnections were automatically excluded from further analysis. Because each participant’s DBS devices operate independently, participant data were aggregated across all nights for each brain hemisphere, resulting in two datasets per participant. Then, each aggregate dataset was epoched into 30 s chunks, with each data epoch corresponding to a single sleep stage label. One label for 30 s of data matches the traditional labeling of sleep physiology. Data epochs were randomly partitioned into either a training set containing 80% of the data or a stratified hold-out test set containing the other 20% of data epochs. Personalized models were trained on the training set and assessed on the hold-out test set for each participant hemisphere, yielding a model for each participant’s left and right hemispheres. Additionally, to analyze model generalization across novel nights, we recapitulated the above analysis while assessing models on a hold-out test set of two randomly chosen nights, while training the models on the remaining nights, in a leave-group-out fashion. These results are provided in Supplementary Fig. 4.

### Model architectures, feature engineering, and model training

Current DBS devices typically leverage spectral power features for classification tasks. Spectral power can encapsulate features related to sleep physiology^18^. However, spectral processing approaches may potentially underutilize information in the underlying time series data streams that are relevant for the sleep classification task. Additionally, raw time series data are utilized for manual AASM sleep scoring^5^. We therefore evaluated three feature processing approaches for electrophysiology: (average) power spectral density power, time-frequency resolved (spectrograms), and time series; and chose established, validated classification models for each feature category.

#### Power spectral density classification

For the average spectral power features, we chose Light Gradient Boosting Machine (LightGBM) and Multi-Layer Perceptron (MLP) because of their relatively simple implementation and wide-ranging utility in supervised classification problems^45^. LightGBM is a gradient-boosting tree-based methodology shown to be effective at sleep staging^29,52^. MLP is a feedforward, fully connected artificial neural network containing two to four hidden layers, and is well-known for generalized classification tasks^26^. The LightGBM and MLP models were trained to predict sleep stages from intracranial power spectral density (PSD) vectors generated for each 30-s epoch. These two models belong to the “spectral” categorization because they are trained on data vectors containing only spectral power information. PSD vectors for each 30 s epoch were generated using Welch’s method with two-second windows and 50% overlap^53^, and truncated to only include frequencies between 0.5 and 60 Hz. Power in each frequency bin was transformed via natural logarithm and standardized across epochs. PSD vectors were calculated for each field potential channel individually, then concatenated together. To address the difference in sample number between sleep stages, class imbalance in the training set was corrected via synthetic minority oversampling technique of minority classes (SMOTE)^54^. Five iterations of Bayesian hyperparameter optimization were performed using five-fold cross-validation on the training set. Hyperparameter optimization for LightGBM was performed over maximum depth, leaves, and number of estimators, for each hemisphere. For MLP, hyperparameter optimization was performed over learning rate, dropout, epoch, and batch size, number of hidden layers (2–5), and hidden layer size (16–384). The scope of hidden layer depth and sizes provides a dynamic range of potentially suitable models for the dimensionality of our feature space (either 120, 240, or 360 features), without placing high prior emphasis on any individual model architecture choice. After identification of hyperparameters, the final model was trained on the entire training set and performance tested on the unseen stratified hold-out test set.

#### Spectrogram image classification

We also include a time-frequency resolved feature category as an intermediate modality between spectral power and continuous raw time series classification. The time-frequency pipeline does not collapse the spectral features across time, but rather analyses the spectral power at individual timesteps in the form of the spectrogram image. We chose AlexNet for “time-frequency” resolved classification^55^. AlexNet is a convolutional neural network consisting of five convolutional layers, followed by three fully connected layers, designed to classify objects in images, here trained on spectrograms.

To transform the field potentials into time-frequency resolved data points, we generated log spectrograms of each 30-s epoch for each field potential stream. Spectrograms were generated using 4 s windows and 50 ms increments, with each window scaled to power spectral density vectors so that all data points are real-valued. Spectrograms for each field potential are stacked, resembling the stacking of color channels common to image classification tasks, and ingested into AlexNet for training and classification^55^. We employed adaptive pooling to address the discrepancy in pixel size from our spectrograms and ImageNet image dimensionality for which AlexNet is designed. To improve model generalization across data epochs and potential overfitting, the training set was augmented via channel shuffling and masking a portion of the frequency data in 15% of the training samples. Class imbalance was addressed by random oversampling of the minority classes. The augmented data was added to the original data, artificially increasing the size of the training set. Bayesian hyperparameter optimization over epoch and batch size, dropout, and learning rate was performed using a random 80/20 stratified split of the training data set into a sub-training and validation set, respectively. Final metrics are reported on the unseen stratified hold-out test set.

#### Time series classification

For time series approaches, models were needed that are capable of capturing temporal features and more sophisticated data statistics. Each index in a time series data epoch corresponds to a measurement of millivolts, and the indices correspond to time points. We tested EEGNet and AttnSleepEnsemble^39,56^. EEGNet is an effective convolutional network architecture designed for feature extraction and classification of electrophysiology time series data^56^. For EEGNet, time series data was preprocessed by splitting the raw field potential data into five band-passed and downsampled time series vectors (similar to^57^. 8th-order butterworth forwards and backwards filters were applied to the raw time series vector for the delta (0.5–4 Hz), theta (4–8 Hz), alpha (8–12 Hz), beta (12–30 Hz), and gamma (30–60 Hz) frequency bands. After bandpassing, each resultant stream was downsampled by a factor of four to a sampling rate of 125 Hz. The resultant bandpass-filtered time series vectors act as surrogate “electrodes” to more closely recapitulate the native (multi-channel) data format for EEGNet. EEGNet hyperparameter optimization was performed over learning rate, epoch, and batch size, number of temporal and depthwise filters, and convolutional factor and kernel sizes.

AttnSleepEnsemble is a customized version of the AttnSleep model^39^. AttnSleep is a convolutional architecture with attention mechanisms, designed to classify sleep stages on a single EEG channel. With three available field potential channels, for this study we employed an ensemble approach with AttnSleep, hereafter called “AttnSleepEnsemble”, where an AttnSleep model is trained on a single channel. For example, one AttnSleep model would be trained on the basal ganglia field potential data, another on the precentral gyrus data, and a final model on the cross-central sulcus data. Each model contributes a single vote for the sleep stage prediction. The majority vote across the individual model predictions is the final prediction for a given data epoch, with tie-breaks defaulting to the deepest sleep stage.

The AttnSleepEnsemble ingested lightly processed time series vectors. We first low-pass the time series at 60 Hz to be consistent with the spectral information provided to other models, and to additionally remove any stimulation artifact. Additionally, to account for the higher sampling rate for the intracranial data compared to typical EEG data (500 Hz vs 100 Hz), we subsequently downsample the time series to 100 Hz sampling rate^39^. We assess performance of AttnSleepEnsemble using “out-of-the-box” (i.e., default) hyperparameters, e.g., kernel window sizes, described in the original manuscript.

For the time domain models, the training set was augmented via the addition of Gaussian noise into the training samples^58^. The augmented training set was added to the original, unaltered training set, thereby doubling the total size of the training data. Class imbalance was then addressed by random oversampling of the minority classes. Final metrics are reported on the stratified hold-out test set. A table of the hyperparameters for each model, and the subsequent ranges sampled for optimization, is provided in Supplementary Fig. 2.

### Assessment of embeddable (Constrained) models

To benchmark implementable sleep-staging techniques, we use the RC + S device as an example of discriminatory features and models that can be currently utilized by DBS devices. Briefly, the RC + S is capable of binary classification (i.e., two-class discrimination) from a linear model using up to four power bands as feature inputs. Previous studies have demonstrated that NREM sleep disruption is significantly correlated with Parkinson’s symptom progression, and that reducing stimulation amplitude during N3 sleep may augment low-frequency neural oscillations (Schreiner et al.^9^; Smyth et al.^21^). Therefore, there is clinical interest to explore stimulation policies specifically targeted towards NREM sleep.

To analyze the performance of device-capable sleep stage discrimination, we assess the performance of embeddable Linear Discriminant Analysis (LDA) models to classify between NREM (N1, N2, and N3) vs other (REM and awake) utilizing the canonical powerbands (delta, alpha plus theta, beta, and low gamma). We refer to data preprocessing and model training pipelines that are limited to current DBS device capabilities as “constrained” (Fig. 1C). Five-fold cross validation for hyperparameter optimization of LDA models was performed over the solver and the tolerance.

We compare both LDA and LightGBM models trained on the natural logarithm of canonical power bands versus the entire PSD vector (Fig. 6). Power bands were calculated as the sum of the power in the relevant frequency bins from the PSD vector. Frequency bins outside of canonical power bands were thus omitted. Preprocessing was performed similarly to the *power spectral density* category above. Although the RC + S device cannot explicitly calculate the logarithm function, linear estimates of the logarithm function can be employed on the system. Including the logarithm transform confers a minor performance enhancement for cortical data (Supplementary Fig. 8), and was included for consistency with the LightGBM methodologies described above.

### Generation and assessment of unsupervised labels for NREM classification

We explored the efficacy of unsupervised clusters as surrogates for sleep stage labels in discriminating NREM from REM and wake stages. We coupled the unsupervised clustering with Linear Discriminant Analysis to assess the clusters' predictive abilities for classical sleep stages. We chose a binary classification schema to analyze the effectiveness of unsupervised approaches for embeddable, i.e., constrained, control policies targeting NREM versus REM and wake (see sections Assessment of embeddable models and Performance of embeddable models). Therefore, the N1-3 stages are consolidated into NREM classification, and REM and wake stages are also aggregated into a single class REM+Wake. We analyze how well the two unsupervised generated clusters map to these two aggregated sleep stage groupings.

Canonical power band data (as described in Assessment of embeddable models) were labeled as either NREM (corresponding to stages N1-3) or other (REM and Wake) (Fig. 8). An LDA model was trained to discriminate these two classes from each other as specified in section Assessment of embeddable models, and tested on a stratified hold-out test set containing NREM vs other labels. We refer to this procedure as the “supervised LDA” approach to NREM vs other classification.

We compare the “supervised LDA” approach with an “unsupervised LDA” approach. In the “unsupervised LDA” approach, the canonical power bands in the training set were stripped of their sleep stage labels. The training set was subsequently clustered using Gaussian Mixture Models (GMMs) with the cluster number hyperparameter set to two^59^. The cluster identity for each data point thus became its class label for model training (e.g., cluster 0 and cluster 1), acting as surrogates for the original, binary sleep stage labels NREM versus REM+Wake, respectively. For consistency, the cluster with higher average delta power was always labeled as cluster 0. An LDA model was trained on the power band training set to discriminate between cluster 0 and cluster 1 with default hyperparameters (singular value decomposition solver and tolerance at 1e-4). The LDA defines a linear boundary for distinguishing between the two unsupervised clusters. We refer to this LDA model as the “unsupervised LDA”. Default hyperparameters for “unsupervised” approach were chosen to adhere to the assumption that no prior information on LDA performance on sleep stage labels is known when training on cluster identity.

However, the “unsupervised LDA” was tested using the same hold-out test set as the “supervised LDA” model. Therefore, the reported results for both the “supervised” and “unsupervised” LDA approaches are on identical hold-out test sets containing sleep stage NREM vs other labels.

In both of these “supervised” and “unsupervised” LDA approaches, training set points were ranked based on their average Euclidean distance to their closest 15 neighbors. The data points within the top 2.5% of average distance to the 15 closest neighbors were excluded; this preprocessing step ensures that a purely noise cluster corresponding to outliers does not appear as a surrogate class label in the ‘unsupervised’ approach^60^. Additionally, no class imbalance correction was performed on the training set, as class imbalance requires sleep stage labels, which are not used when training the ‘unsupervised’ approach.

## Supplementary information

Sleep Classification - Supplement Updated

## Data Availability

The datasets generated and/or analysed during the current study are not publicly available due to Personal Health Information reasons, but are available from the corresponding author on reasonable request.

## Code availability

The underlying code for this study, and training/validation datasets, is not publicly available but may be made available to qualified researchers on reasonable request from the corresponding author.
