# Supplementary material for: Personalized supervised and unsupervised intracranial sleep decoding during deep brain stimulation
Source: NPJ Digit Med. 2026 Jan 22;9:550. doi: 10.1038/s41746-026-02368-0 (PMC13376363; doi:10.1038/s41746-026-02368-0)
Supplement: Supplementary file 1 — Sleep Classification - Supplement Updated [file 41746_2026_2368_MOESM1_ESM.pdf]

Supplementary Figures

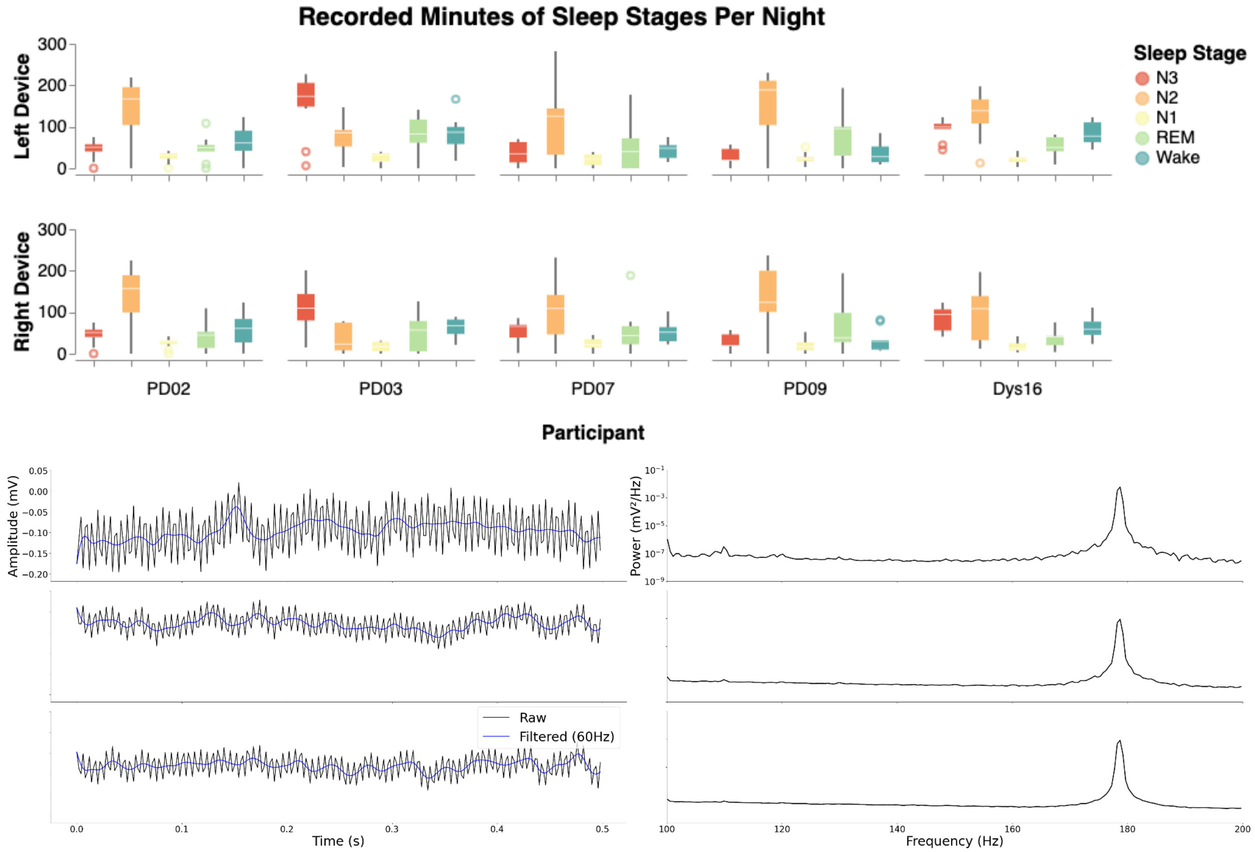

Supplementary Figure 1. (Top) Box plots depicting the distributions of recorded time in each sleep stage across nights. Colors indicate distinct sleep stages, with columns corresponding to participants and rows corresponding to each brain hemisphere. (Bottom) Left: Representative half-second traces of the raw field potentials for PD03L device, with a 60 Hz lowpassed trace overlaid. Right: Power spectral density plots of the stimulation artifact. Top row to bottom row: Basal ganglia, Cross-central sulcus, Pre-central gyrus streams. Similar stimulation artifacts are seen for other devices.

Model Hyperparameters												
	Type	Values Range	02L	02R	03L	03R	07L	07R	09L	09R	16L	16R
**AlexNet**												
batch_size	categorical	[64,128,256]	64	128	64	64	64	256	64	256	128	128
criterion	fixed	CrossEntropy	CrossEntropy	CrossEntropy	CrossEntropy	CrossEntropy	CrossEntropy	CrossEntropy	CrossEntropy	CrossEntropy	CrossEntropy	CrossEntropy
dropout	continuous	[0.01, 0.5]	0.17	0.34	0.14	0.39	0.15	0.46	0.24	0.18	0.46	0.01
epochs	categorical	[30,50,80,120]	120	80	80	30	120	80	50	120	50	120
lr	continuous	[0.00005, 0.0005]	8.53e-05	3.70e-04	8.60e-05	2.84e-04	1.47e-04	8.41e-05	2.83e-04	2.19e-04	8.40e-05	5.09e-05
optimizer	fixed	adam	adam	adam	adam	adam	adam	adam	adam	adam	adam	adam
**EEGNet**												
D	categorical	[1,2,3,4]	2	1	1	4	3	4	3	2	3	3
F1	categorical	[5,10,15]	15	15	15	10	15	10	15	10	5	15
F2	categorical	[5,10,15]	10	5	10	15	15	15	10	10	10	10
batch_size	categorical	[64,128,256]	256	128	128	64	128	128	256	256	128	64
chunk_size	fixed	3750	3750	3750	3750	3750	3750	3750	3750	3750	3750	3750
criterion	fixed	CrossEntropy	CrossEntropy	CrossEntropy	CrossEntropy	CrossEntropy	CrossEntropy	CrossEntropy	CrossEntropy	CrossEntropy	CrossEntropy	CrossEntropy
dropout	continuous	[0.01, 0.5]	0.25	0.36	0.44	0.46	0.15	0.40	0.15	0.21	0.30	0.14
epochs	categorical	[30,50,80,120]	50	50	30	120	30	80	120	120	30	50
kernel_1	categorical	[32,64,128,256]	128	256	64	32	128	64	64	128	64	128
kernel_2	categorical	[4,8,16,32,64]	64	32	32	4	4	64	64	64	32	8
lr	continuous	[0.00005, 0.0005]	2.42e-04	2.36e-04	3.36e-04	2.40e-04	2.22e-04	4.39e-04	4.33e-04	1.46e-04	7.19e-05	9.31e-05
optimizer	fixed	adam	adam	adam	adam	adam	adam	adam	adam	adam	adam	adam
**LDA**												
solver	categorical	["svd","lsqr","eigen"]	-	-	-	-	-	-	-	-	-	-
tol	categorical	[0.0001,0.001,0.01,0.1]	-	-	-	-	-	-	-	-	-	-
**LightGBM**												
max_depth	categorical	[2,4,8,12,14,16,20]	12	2	8	2	20	14	20	12	4	8
max_depth	categorical	[2,4,8,12,14,16,20]	4	2	8	2	20	14	20	12	4	8
max_depth	categorical	[2,4,8,12,14,16,20]	12	2	4	2	20	14	20	12	4	8
max_depth	categorical	[2,4,8,12,14,16,20]	4	2	4	2	20	14	20	12	4	8
n_estimators	categorical	[100,200,300,400,500,600,800,900]	500	100	800	100	400	600	300	900	100	300
n_estimators	categorical	[100,200,300,400,500,600,800,900]	300	100	800	100	400	600	300	900	100	300
n_estimators	categorical	[100,200,300,400,500,600,800,900]	500	100	600	100	400	600	300	900	100	300
n_estimators	categorical	[100,200,300,400,500,600,800,900]	300	100	600	100	400	600	300	900	100	300
num_leaves	categorical	[3,5,7,15,31,63,127]	127	3	7	5	3	5	15	31	5	3
num_leaves	categorical	[3,5,7,15,31,63,127]	63	3	7	5	3	5	15	31	5	3
num_leaves	categorical	[3,5,7,15,31,63,127]	127	3	15	5	3	5	15	31	5	3
num_leaves	categorical	[3,5,7,15,31,63,127]	63	3	15	5	3	5	15	31	5	3
**MLP**												
activation	fixed	leaky_relu	leaky_relu	leaky_relu	leaky_relu	leaky_relu	leaky_relu	leaky_relu	leaky_relu	leaky_relu	leaky_relu	leaky_relu
batch_size	categorical	[64,128,256]	256	256	128	64	256	128	256	256	64	128
criterion	fixed	CrossEntropy	CrossEntropy	CrossEntropy	CrossEntropy	CrossEntropy	CrossEntropy	CrossEntropy	CrossEntropy	CrossEntropy	CrossEntropy	CrossEntropy
dropout	continuous	[0.01, 0.5]	0.01	0.19	0.12	0.45	0.05	0.43	0.21	0.34	0.12	0.08
epochs	categorical	[10,15,20,30]	10	15	15	15	20	30	10	10	30	15
hidden_size	categorical	[16,32,64,96,128,256,384]	32	384	16	64	256	256	128	128	128	256
lr	continuous	[0.0001, 0.1]	2.37e-02	6.53e-02	2.80e-02	6.78e-02	1.83e-02	8.78e-02	5.95e-02	1.72e-02	7.14e-02	9.10e-02
n_layer	categorical	[2,3,4,5]	5	4	3	5	5	5	5	5	4	2
optimizer	fixed	adam	adam	adam	adam	adam	adam	adam	adam	adam	adam	adam
regularization	fixed	L2	L2	L2	L2	L2	L2	L2	L2	L2	L2	L2

Supplementary Figure 2: Table summarizing the hyperparameter space optimized over for each model during Bayesian Optimization training. Models were trained and optimized over the provided hyperparameter space for each participant hemisphere (L vs R) and field potential streams (CTX+BG, BG, CS) individually. The “Type” column states the nature of the hyperparameter search space. “Fixed” indicates the value of the hyperparameter in the “Values Range” column was unchanging across all models. “Categorical” indicates a pre-specified set of hyperparameter values that was optimized over. (Caption continues on next page)

Supplementary Figure 2 Continued: “Continuous” indicates a range of possible values (inclusive of boundaries) which was optimized over for each model. Example hyperparameters are included for the models depicted Figure 3.

Model Accuracy										
	02L	02R	03L	03R	07L	07R	09L	09R	16L	16R
AlexNet	0.79	0.77	0.70	0.68	0.79	0.74	0.72	0.75	0.62	0.63
AttnSleepEnsemble	0.72	0.71	0.68	0.72	0.75	0.73	0.76	0.76	0.66	0.67
EEGNet	0.77	0.80	0.71	0.61	0.66	0.69	0.73	0.71	0.63	0.65
LightGBM	0.85	0.83	0.76	0.78	0.82	0.82	0.80	0.79	0.71	0.74
MLP	0.80	0.80	0.73	0.72	0.79	0.81	0.72	0.75	0.64	0.64

Supplementary Figure 3. Model accuracies, as reported by the hold-out test set, without applying class imbalance correction and data augmentation when training on the test set.

A

Accuracy by Brain Region

	02L	02R	03L	03R	07L	07R	09L	09R	16L	16R
**AlexNet**										
BG	0.70	0.69	0.72	0.63	0.57	0.61	0.41	0.62	0.47	0.47
CS	0.73	0.76	0.65	0.62	0.59	0.73	0.80	0.77	0.55	0.43
CTX+BG	0.77	0.76	0.73	0.65	0.64	0.68	0.82	0.78	0.60	0.57
**AttnSleepEnsemble**										
BG	0.70	0.65	0.73	0.61	0.41	0.44	0.44	0.67	0.50	0.43
CS	0.78	0.72	0.63	0.69	0.60	0.73	0.80	0.78	0.61	0.48
CTX+BG	0.78	0.78	0.68	0.72	0.62	0.69	0.81	0.82	0.61	0.57
**EEGNet**										
BG	0.48	0.57	0.67	0.64	0.45	0.36	0.21	0.55	0.42	0.44
CS	0.70	0.69	0.68	0.66	0.59	0.66	0.74	0.73	0.60	0.53
CTX+BG	0.76	0.72	0.69	0.65	0.60	0.70	0.78	0.75	0.63	0.60
**LightGBM**										
BG	0.64	0.68	0.74	0.64	0.61	0.60	0.43	0.72	0.57	0.52
CS	0.81	0.80	0.72	0.75	0.67	0.75	0.80	0.81	0.66	0.68
CTX+BG	0.82	0.80	0.79	0.77	0.69	0.76	0.85	0.80	0.64	0.67
**MLP**										
BG	0.55	0.68	0.55	0.61	0.56	0.63	0.46	0.61	0.41	0.40
CS	0.75	0.74	0.56	0.58	0.67	0.63	0.74	0.76	0.62	0.61
CTX+BG	0.80	0.71	0.73	0.51	0.71	0.71	0.65	0.73	0.62	0.47

B

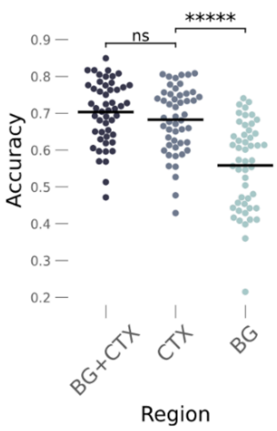

C

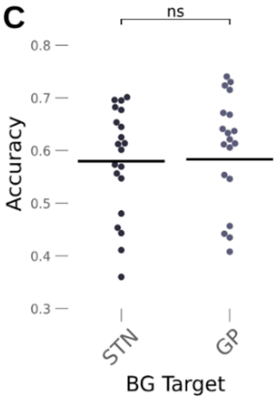

D

Accuracy for Two Stage Classification

	Power Spectral Density										*Power Bands*									
	02L	02R	03L	03R	07L	07R	09L	09R	16L	16R	02L	02R	03L	03R	07L	07R	09L	09R	16L	16R
**LDA**																				
BG	0.79	0.81	0.91	0.82	0.79	0.82	0.56	0.84	0.84	0.68	0.75	0.82	0.88	0.79	0.76	0.81	0.70	0.86	0.84	0.67
CS	0.87	0.87	0.91	0.87	0.85	0.91	0.90	0.90	0.87	0.87	0.85	0.85	0.90	0.87	0.85	0.91	0.87	0.88	0.87	0.87
CTX+BG	0.88	0.90	0.92	0.88	0.79	0.86	0.88	0.90	0.86	0.86	0.87	0.89	0.90	0.87	0.84	0.91	0.86	0.90	0.87	0.87
**LightGBM**																				
BG	0.79	0.84	0.90	0.79	0.82	0.84	0.57	0.84	0.85	0.75	0.78	0.80	0.88	0.80	0.76	0.76	0.56	0.84	0.80	0.63
CS	0.90	0.88	0.92	0.88	0.84	0.88	0.91	0.91	0.87	0.88	0.86	0.85	0.89	0.88	0.80	0.87	0.88	0.88	0.86	0.86
CTX+BG	0.89	0.91	0.92	0.88	0.86	0.90	0.91	0.90	0.87	0.88	0.86	0.88	0.91	0.88	0.82	0.86	0.90	0.90	0.87	0.87

E

Accuracy by Brain Region

	02L	02R	03L	03R	07L	07R	09L	09R	16L	16R
**Supervised LDA**										
BG	0.78	0.82	0.83	0.81	0.82	0.86	0.64	0.82	0.81	0.74
CS	0.86	0.86	0.87	0.85	0.86	0.89	0.85	0.84	0.84	0.85
CTX+BG	0.86	0.90	0.88	0.89	0.88	0.89	0.85	0.87	0.85	0.86
**Unsupervised LDA**										
BG	0.69	0.68	0.63	0.70	0.59	0.87	0.32	0.84	0.36	0.64
CS	0.87	0.87	0.90	0.87	0.89	0.90	0.83	0.69	0.87	0.87
CTX+BG	0.87	0.78	0.74	0.86	0.90	0.90	0.68	0.83	0.87	0.86

Supplementary Figure 4. Model accuracies when using a leave-k-groups-out test set. For each device, two nights of sleep data were held-out as the test set, on which the accuracy was assessed. (A) Recapitulation of the analysis in Figure 5Figure 6, where each model was assessed on the corresponding brain region for each device. (B-C) Comparison of model performance when partitioned by data preprocessing and model choice (B) and BG target (C). We note that statistical significance when comparing model results for BG vs CS (Linear Mixed Model [LMM], using each unique grouping of (model, hemisphere) as the random effect; $p<<1e-5$; $n=50$), but significance was not achieved for CS vs CTX+BG ($p=0.2$; $n=50$). (D) Recapitulation of the two state classification contingency, described in section 4.4.1 and Table 2. (E) Recapitulation of the unsupervised classification approach described in section 4.4.2 and Figure 6Figure 7.

Model Accuracy By Hemisphere	02	03	07	09	16
**AlexNet**					
Both	0.78	0.76	0.80	0.73	0.62
Left	0.79	0.70	0.79	0.72	0.62
Right	0.77	0.68	0.74	0.75	0.63
**AttnSleepEnsemble**					
Both	0.77	0.75	0.73	0.79	0.64
Left	0.72	0.68	0.75	0.76	0.66
Right	0.72	0.72	0.73	0.76	0.67
**EEGNet**					
Both	0.78	0.70	0.63	0.72	0.61
Left	0.77	0.71	0.66	0.73	0.63
Right	0.80	0.61	0.70	0.71	0.65
**LightGBM**					
Both	0.86	0.82	0.86	0.85	0.73
Left	0.85	0.76	0.82	0.80	0.71
Right	0.83	0.78	0.82	0.79	0.74
**MLP**					
Both	0.82	0.79	0.81	0.75	0.63
Left	0.80	0.73	0.79	0.72	0.64
Right	0.80	0.72	0.81	0.75	0.64

Supplementary Figure 5. Model accuracies as compared across hemispheres, or aggregated across hemispheres (‘Both’). When aggregating data across hemispheres, epochs when data was only present in a single hemisphere (e.g. the contralateral device had a temporary disconnection) were removed from the dataset.

Model Sensitivity

	02L	02R	03L	03R	07L	07R	09L	09R	16L	16R
**AlexNet**										
N1	0.38	0.43	0.00	0.00	0.07	0.32	0.03	0.22	0.15	0.12
N2	0.85	0.88	0.44	0.22	0.76	0.71	0.88	0.84	0.56	0.71
N3	0.70	0.69	0.86	0.89	0.58	0.83	0.23	0.53	0.80	0.70
REM	0.57	0.44	0.60	0.52	0.79	0.86	0.66	0.71	0.66	0.68
Wake	0.88	0.92	0.72	0.68	0.73	0.73	0.80	0.79	0.42	0.46
**AttnSleepEnsemble**										
N1	0.25	0.30	0.08	0.13	0.00	0.00	0.13	0.07	0.04	0.12
N2	0.90	0.88	0.38	0.18	0.86	0.67	0.85	0.76	0.80	0.83
N3	0.63	0.52	0.87	0.90	0.19	0.79	0.13	0.33	0.66	0.42
REM	0.54	0.61	0.68	0.46	0.83	0.91	0.73	0.85	0.77	0.77
Wake	0.86	0.88	0.66	0.73	0.76	0.73	0.82	0.71	0.38	0.38
**EEGNet**										
N1	0.58	0.66	0.32	0.31	0.47	0.16	0.30	0.04	0.27	0.38
N2	0.66	0.70	0.56	0.29	0.68	0.62	0.74	0.72	0.53	0.60
N3	0.92	0.94	0.83	0.83	0.48	0.85	0.70	0.65	0.81	0.79
REM	0.67	0.55	0.60	0.54	0.85	0.90	0.74	0.77	0.73	0.82
Wake	0.86	0.84	0.81	0.82	0.69	0.75	0.95	0.81	0.38	0.39
**LightGBM**										
N1	0.48	0.55	0.12	0.13	0.27	0.32	0.20	0.37	0.15	0.17
N2	0.88	0.80	0.58	0.45	0.73	0.85	0.89	0.86	0.73	0.75
N3	0.85	0.87	0.88	0.89	0.79	0.93	0.68	0.70	0.81	0.79
REM	0.72	0.76	0.77	0.65	0.85	0.89	0.77	0.80	0.79	0.81
Wake	0.87	0.89	0.82	0.86	0.92	0.78	0.83	0.81	0.49	0.51
**MLP**										
N1	0.53	0.62	0.20	0.13	0.60	0.42	0.47	0.15	0.19	0.46
N2	0.82	0.85	0.47	0.02	0.71	0.82	0.58	0.75	0.52	0.72
N3	0.88	0.74	0.81	0.86	0.77	0.80	0.88	0.81	0.80	0.52
REM	0.61	0.70	0.63	0.08	0.91	0.82	0.68	0.75	0.74	0.90
Wake	0.91	0.86	0.82	0.98	0.80	0.78	0.87	0.79	0.32	0.42

Supplementary Figure 6. Model sensitivity for each individual sleep stage across all participant hemispheres.

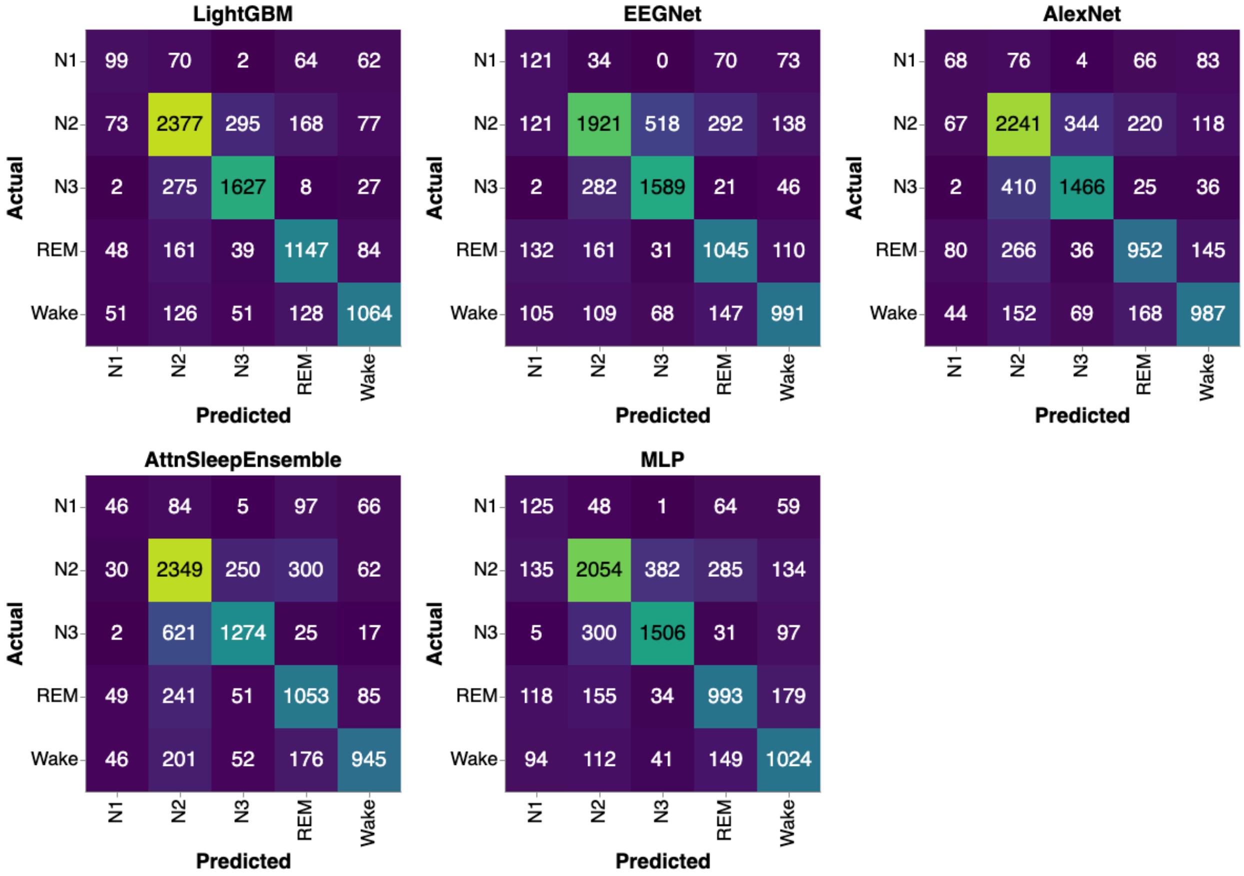

Supplementary Figure 7. Confusion matrices of each model's performance on all three channels (CTX+BG), aggregated across all participant hemispheres.

LDA Accuracy by Processing Method

	02L	02R	03L	03R	07L	07R	09L	09R	16L	16R
**Log Transform**										
BG	0.78	0.82	0.83	0.81	0.82	0.86	0.64	0.82	0.81	0.74
CS	0.86	0.86	0.87	0.85	0.86	0.89	0.85	0.84	0.84	0.85
CTX+BG	0.86	0.90	0.88	0.89	0.88	0.89	0.85	0.87	0.85	0.86
**No Log Transform**										
BG	0.75	0.72	0.76	0.80	0.76	0.76	0.64	0.81	0.82	0.72
CS	0.84	0.86	0.88	0.84	0.82	0.86	0.84	0.85	0.84	0.85
CTX+BG	0.86	0.87	0.85	0.86	0.89	0.89	0.83	0.84	0.83	0.85

Supplementary Figure 8. Table comparing hold-out test set accuracies on NREM vs Wake+REM classification for Linear Discriminant Models. *Log Transform* indicates the power band data points underwent a natural logarithm transformation prior to z-scoring and training. *No Log Transform* indicates that the raw power band data was z-scored and used for training.

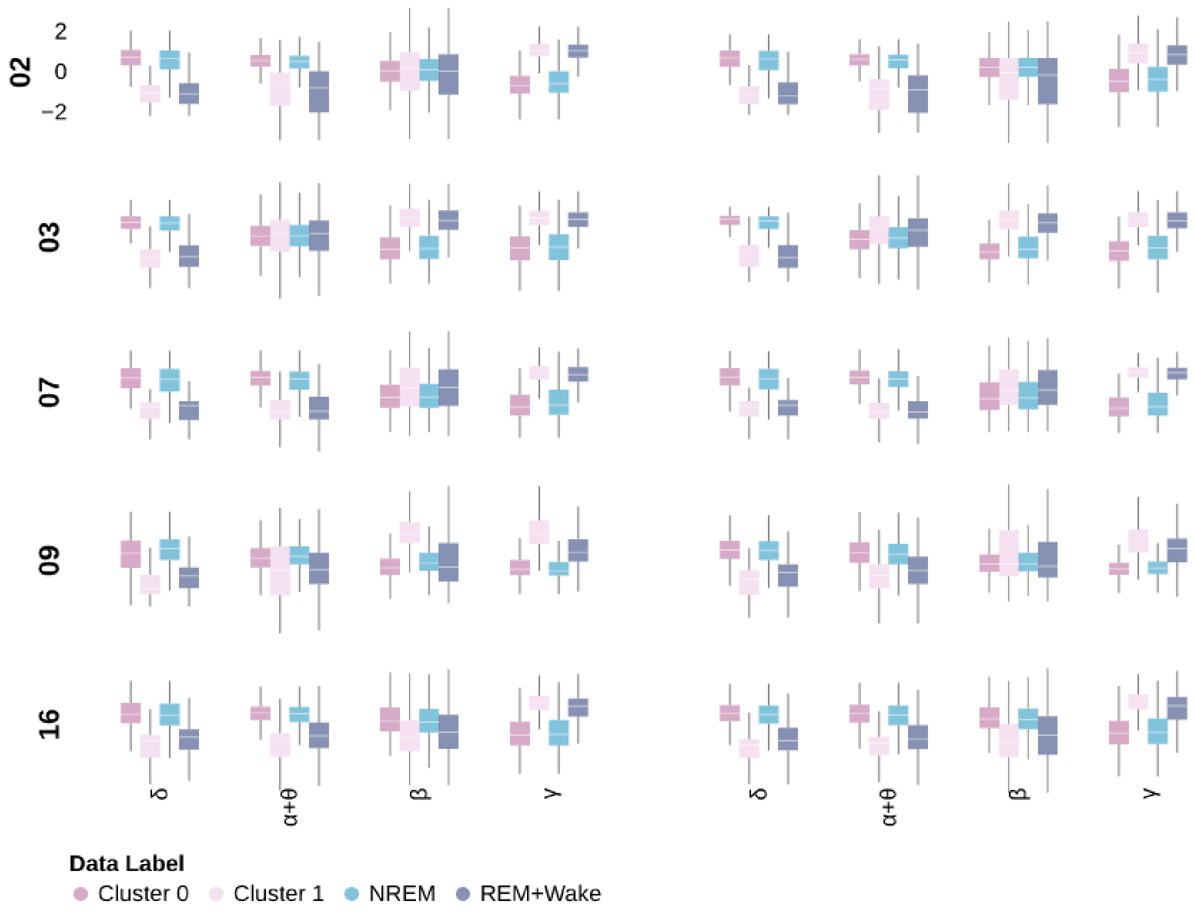

Supplementary Figure 9. Distributions of Supervised vs Unsupervised training data labels, as described in Figure 6A, for all participants. Left grouping of columns corresponds to the left hemisphere, while right grouping corresponds to the right hemisphere.
